# 2-Amino-4-methyl-6-oxo-3,6-dihydro­pyrimidin-1-ium perchlorate–2-amino-6-methyl­pyrimidin-4(1*H*)-one–water (1/1/1)

**DOI:** 10.1107/S1600536811034106

**Published:** 2011-08-27

**Authors:** Kamel Kaabi, Maher El Glaoui, Valeria Ferretti, Matthias Zeller, Cherif Ben Nasr

**Affiliations:** a Laboratoire de Chimie des Matériaux, Faculté des Sciences de Bizerte, 7021 Zarzouna, Tunisia; b Chemistry Department and Centro di Strutturistica Diffrattometrica, University of Ferrara, Via L. Borsari 46, I-44121 Ferrara, Italy; c Youngstown State University, Department of Chemistry, One University Plaza, Youngstown, Ohio 44555-3663, USA

## Abstract

In the title compound, C_5_H_8_N_3_O^+^·ClO_4_^−^·C_5_H_7_N_3_O·H_2_O, each perchlorate anion is paired with a protonated cationic 2-amino-6-methyl­pyrimidin-4(1*H*)-one and another non-protonated entity of the same organic pyrimidinone. The crystal structure is stabilized by N—H⋯O_org_, N—H⋯O_water_, N—H⋯O_ClO4_, O—H⋯O_ClO4_, N—H⋯N and C—H⋯O_ClO4_ hydrogen bonds between the anions, organic entities and water mol­ecules. Inter­molecular π–π stacking inter­actions between neighbouring organic rings are observed with a face-to-face distance of 3.776 (2) Å, and O—H⋯O hydrogen bonds link the perchlorate anions and the water mol­ecules into chains along the *b*-axis direction. The perchlorate anion and the inter­stitial water mol­ecule are disordered over two mutually incompatible positions with a common occupancy ratio of 0.678 (16):0.322 (16).

## Related literature

For general background to perchlorate salts with organic cations, see: Czarnecki *et al.* (1994 ▶); Czupinski *et al.* (2002 ▶, 2006 ▶). For enamine-imino resonance, see: Oueslati *et al.* (2007 ▶). For π–π stacking inter­actions, see: Janiak (2000 ▶).
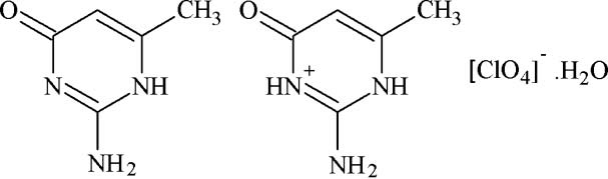


## Experimental

### 

#### Crystal data

C_5_H_8_N_3_O^+^·ClO_4_^−^·C_5_H_7_N_3_O·H_2_O
*M* *_r_* = 368.75
Monoclinic, 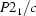

*a* = 10.3669 (3) Å
*b* = 10.4342 (3) Å
*c* = 15.0780 (5) Å
β = 92.751 (2)°
*V* = 1629.11 (9) Å^3^
*Z* = 4
Mo *K*α radiation
μ = 0.28 mm^−1^
*T* = 295 K
0.30 × 0.15 × 0.12 mm

#### Data collection

Nonius Kappa CCD diffractometer
7792 measured reflections
4739 independent reflections
2765 reflections with *I* > 2σ(*I*)
*R*_int_ = 0.027

#### Refinement

*R*[*F* ^2^ > 2σ(*F* ^2^)] = 0.059
*wR*(*F* ^2^) = 0.184
*S* = 1.02
4739 reflections
279 parameters
64 restraints
H atoms treated by a mixture of independent and constrained refinement
Δρ_max_ = 0.38 e Å^−3^
Δρ_min_ = −0.47 e Å^−3^

### 

Data collection: Kappa CCD server software (Nonius, 1997 ▶); cell refinement: *DENZO-SMN* (Otwinowski & Minor, 1997 ▶); data reduction: *DENZO-SMN*; program(s) used to solve structure: *SIR97* (Altomare *et al.*,1999 ▶); program(s) used to refine structure: *SHELXL97* (Sheldrick, 2008 ▶); molecular graphics: *ORTEPIII* (Burnett & Johnson, 1996 ▶); software used to prepare material for publication: *SHELXL97*, *PARST* (Nardelli, 1983 ▶, 1995 ▶), *WinGX* (Farrugia, 1999 ▶).

## Supplementary Material

Crystal structure: contains datablock(s) global, I. DOI: 10.1107/S1600536811034106/bx2369sup1.cif

Structure factors: contains datablock(s) I. DOI: 10.1107/S1600536811034106/bx2369Isup2.hkl

Supplementary material file. DOI: 10.1107/S1600536811034106/bx2369Isup3.cml

Additional supplementary materials:  crystallographic information; 3D view; checkCIF report

## Figures and Tables

**Table 1:** Hydrogen-bond geometry (Å, °)

*D*—H⋯*A*	*D*—H	H⋯*A*	*D*⋯*A*	*D*—H⋯*A*
O1*WA*—H2*WA*⋯O2	0.82 (2)	2.26 (6)	3.07 (4)	169 (16)
C5—H5*B*⋯O3′	0.96	2.55	3.509 (6)	173
N5—H5⋯O1*WB*	0.86	1.95	2.797 (8)	166
O1*WB*—H2*WB*⋯O4′	0.82 (2)	2.30 (4)	3.071 (14)	159 (6)
N1—H1⋯N4^i^	0.86	1.98	2.839 (2)	174
N3—H3*B*⋯O6^i^	0.86	1.93	2.787 (2)	178
N6—H6*A*⋯O5^i^	0.86	2.05	2.895 (2)	168
N2—H2⋯O6^ii^	0.86	1.84	2.6560 (18)	158
N3—H3*A*⋯O5^ii^	0.86	2.24	3.0363 (18)	154
N6—H6*B*⋯O1^iii^	0.86	2.35	3.095 (17)	145
C3—H3⋯O1^iv^	0.93	2.56	3.466 (17)	166

Symmetry codes: (i) 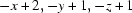; (ii) 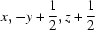; (iii) 
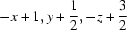
; (iv) 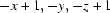.

**Fig. 1.**
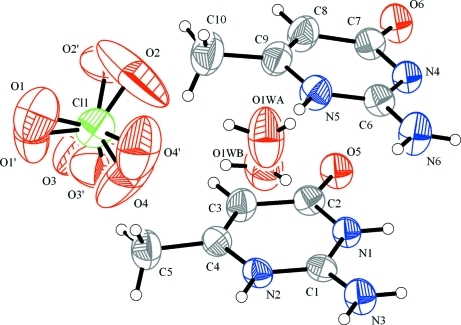
A view of the title compound, showing 50% probability displacement ellipsoids, arbitrary spheres for the H atoms, and the atom numbering scheme.

**Fig. 2.**
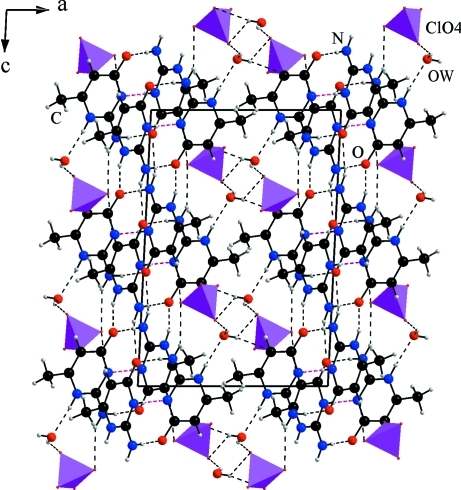
Packing of the title compound viewed down the b axis, showing the hydrogen bonding scheme between the water molecules, perchlorate anions and organic entities. Disorder of perchlorate anions and of water molecules is omitted for clarity.

**Fig. 3.**
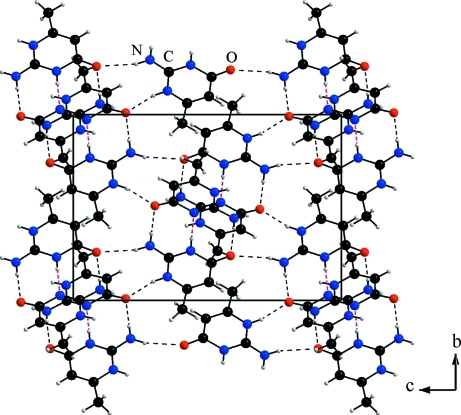
Crystal packing arrangement showing the hydrogen bonding scheme between the organic entities. Hydrogen bonds are denoted by dotted lines. Disorder of perchlorate anions and of water molecules is omitted for clarity.

## supplementary crystallographic information

### Comment

Perchlorate salts containing organic cations have been studied extensively in recent years owing to some of their interesting properties such as *e.g.* ferroelectric and dielectric behaviour (Czarnecki *et al.*, 1994; Czupinski *et al.*, 2002; Czupinski *et al.*, 2006). Here, we report the synthesis and the crystal structure of one such compound, (C_5_H_8_N_3_O)(C_5_H_7_N_3_O)ClO_4_.H_2_O.

The crystal structure of the title compound (Fig.1) contains one perchlorate anion, one water molecule, and two 2-amino-6-methylpyrimidin-4(1*H*)-one molecules. One of these molecules is protonated at the nitrogen atom of the six membered ring, thus formally changing the molecule into a 2-amino-4-methyl-6-oxo-3,6-dihydropyrimidin-1-ium cation. The atomic arrangement of (C_5_H_8_N_3_O)(C_5_H_7_N_3_O)ClO_4_.H_2_O can be divided into an organic and an inorganic part. The inorganic section is composed of chains of [ClO_4_]^-^ tetrahedra and water molecules that extend along the *b* axis direction, held together by O_water_—H···O(ClO_4_) hydrogen bonds. Two such chains cross the unit cell at *z* = (2n +1)/4 and *x* = 0.5 (Fig. 2, Table 1). The organic groups are located between these chains and connect to them through N—H···O_water_, N—H···O(ClO_4_) and C—H···O(ClO_4_) hydrogen bonds to form a three dimensional infinite network (Fig. 3, Table 1). Of the hydrogen bonds, one is bifurcted: O1WA—H2WA···(O2, O4) (Fig. 2, Table 1). The organic entities are associated with each other *via* N—H···O_org_ and N—H···N hydrogen bonds (Fig. 3, Table 1). Intermolecular π-π stacking interactions between neighbouring organic rings are observed with a face-to-face distance of 3.776 (2) Å, less than 3.8 Å, the maximum regarded as relevant for π-π interactions (Janiak, 2000).

The C—N bond distances of the NH_2_ groups, N3—C1 and N6—C6, are 1.311 (2) and 1.327 (2) Å, respectively, which is short for a C—N single bond, but still not quite as contracted as one would expect for a fully established C=N double bond. These bond length features are consistent with an imino resonance form as it is commonly found for C—N single bonds involving *sp*^2^ hybridized C and N atoms (Oueslati *et al.*, 2007). The distance values of C2—O5 [1.233 (2) Å] and C7—O6 [1.260 (2) Å] clearly indicate two C=O double bonds. This confirms that the first step of the formation of the title compound consists in the tautomerization of the starting material 2-amino-4-hydroxy-6-methylpyrimidine into 2-amino-6-methylpyrimidin-4(1*H*)-one.

### Experimental

An aqueous solution of Cu(ClO_4_)_2_ (1 mmol, 0.263 g) was added dropwise to a solution of 2-amino-4-hydroxy-6-methylpyrimidine (1 mmol, 0.125 g) in ethanol. The resultant mixture was evaporated at room temperature. Crystals of the title compound, which remained stable under normal conditions of temperature and humidity, were isolated after several days and subjected to X-ray diffraction analysis (yield 56%).

### Refinement

Reflections (1 1 0), (1 0 0), (-1 0 2), (0 0 2), (0 1 1), (-1 1 1) and (-1 1 2) were obscured by the beamstop and were omitted from the refinement. The oxygen atoms of the perchlorate ion were refined as disordered over two mutually exclusive sets of positions with a refined occupancy ratio of 0.678 (16) to 0.322 (16) for the two orientations. Associated with the perchlorate disorder is disorder of a water molecule, which is distributed over two positions in the same ratio as the anions. All Cl—O bond distances and O···O distances within each disordered moiety were restrained to be eadch the same within a standard deviation of 0.02 Å. C—H and N—H hydrogen atoms were placed in calculated positions with C—H distances of 0.93 and 0.96 Å and N—H distances of 0.86 Å. The the water hydrogen atom postitions were refined with O—H distance restraints of 0.82 (2) Å and H···H distance restraints within each water molecule of 1.35 (2) Å. *U*_iso_(H) values of all H atoms were constrained to 1.2 (amine, C—H) or 1.5 (CH_3_, O—H) times *U*_eq_ of the respective parent atom.

### Crystal data

**Table:**

C_5_H_8_N_3_O^+^·ClO_4_^−^·C_5_H_7_N_3_O·H_2_O	*F*(000) = 768
*M**_r_* = 368.75	*D*_x_ = 1.503 Mg m^−^^3^
Monoclinic, *P*2_1_/*c*	Mo *K*α radiation, λ = 0.71073 Å
Hall symbol: -P 2ybc	Cell parameters from 7792 reflections
*a* = 10.3669 (3) Å	θ = 2.0–30.0°
*b* = 10.4342 (3) Å	µ = 0.28 mm^−^^1^
*c* = 15.0780 (5) Å	*T* = 295 K
β = 92.751 (2)°	Prismatic, colourless
*V* = 1629.11 (9) Å^3^	0.30 × 0.15 × 0.12 mm
*Z* = 4	

### Data collection

**Table:**

Nonius Kappa CCD diffractometer	2765 reflections with *I* > 2σ(*I*)
Radiation source: fine-focus sealed tube	*R*_int_ = 0.027
graphite	θ_max_ = 30.1°, θ_min_ = 3.9°
φ scans and ω scans	*h* = −14→14
7792 measured reflections	*k* = −13→14
4739 independent reflections	*l* = −21→21

### Refinement

**Table:**

Refinement on *F*^2^	Secondary atom site location: difference Fourier map
Least-squares matrix: full	Hydrogen site location: inferred from neighbouring sites
*R*[*F*^2^ > 2σ(*F*^2^)] = 0.059	H atoms treated by a mixture of independent and constrained refinement
*wR*(*F*^2^) = 0.184	*w* = 1/[σ^2^(*F*_o_^2^) + (0.1048*P*)^2^ + 0.0794*P*] where *P* = (*F*_o_^2^ + 2*F*_c_^2^)/3
*S* = 1.02	(Δ/σ)_max_ = 0.001
4739 reflections	Δρ_max_ = 0.38 e Å^−^^3^
279 parameters	Δρ_min_ = −0.47 e Å^−^^3^
64 restraints	Extinction correction: *SHELXL*, Fc^*^=kFc[1+0.001xFc^2^λ^3^/sin(2θ)]^-1/4^
Primary atom site location: structure-invariant direct methods	Extinction coefficient: 0.033 (6)

### Special details

**Table:**

Geometry. All e.s.d.'s (except the e.s.d. in the dihedral angle between two l.s. planes) are estimated using the full covariance matrix. The cell e.s.d.'s are taken into account individually in the estimation of e.s.d.'s in distances, angles and torsion angles; correlations between e.s.d.'s in cell parameters are only used when they are defined by crystal symmetry. An approximate (isotropic) treatment of cell e.s.d.'s is used for estimating e.s.d.'s involving l.s. planes.
Refinement. Refinement of *F*^2^ against ALL reflections. The weighted *R*-factor *wR* and goodness of fit *S* are based on *F*^2^, conventional *R*-factors *R* are based on *F*, with *F* set to zero for negative *F*^2^. The threshold expression of *F*^2^ > σ(*F*^2^) is used only for calculating *R*-factors(gt) *etc*. and is not relevant to the choice of reflections for refinement. *R*-factors based on *F*^2^ are statistically about twice as large as those based on *F*, and *R*- factors based on ALL data will be even larger.

### Fractional atomic coordinates and isotropic or equivalent isotropic displacement parameters (Å^2^)

**Table:**

	*x*	*y*	*z*	*U*_iso_*/*U*_eq_	Occ. (<1)
O5	0.98648 (14)	0.24064 (14)	0.41381 (8)	0.0481 (4)	
N1	0.98287 (15)	0.26652 (14)	0.56248 (9)	0.0370 (4)	
H1	1.0315	0.3329	0.5589	0.044*	
N2	0.87048 (15)	0.12750 (14)	0.64911 (9)	0.0386 (4)	
H2	0.8495	0.1022	0.7007	0.046*	
N3	0.98691 (18)	0.29455 (16)	0.71393 (10)	0.0489 (4)	
H3A	0.9635	0.2717	0.7656	0.059*	
H3B	1.0363	0.3600	0.7086	0.059*	
C1	0.94722 (17)	0.22960 (16)	0.64336 (11)	0.0357 (4)	
C2	0.94490 (18)	0.20263 (17)	0.48438 (11)	0.0384 (4)	
C3	0.85884 (19)	0.09806 (19)	0.49410 (12)	0.0452 (5)	
H3	0.8263	0.0545	0.4441	0.054*	
C4	0.82387 (18)	0.06148 (17)	0.57560 (12)	0.0407 (4)	
C5	0.7351 (2)	−0.0476 (2)	0.59327 (15)	0.0579 (6)	
H5A	0.7149	−0.0929	0.5390	0.087*	
H5B	0.6569	−0.0151	0.6165	0.087*	
H5C	0.7763	−0.1048	0.6357	0.087*	
Cl1	0.32526 (6)	0.13475 (6)	0.68097 (4)	0.0630 (2)	
O1	0.2053 (11)	0.0786 (17)	0.6977 (12)	0.098 (5)	0.322 (16)
O2	0.308 (2)	0.2428 (18)	0.6303 (18)	0.206 (11)	0.322 (16)
O3	0.3952 (12)	0.0439 (15)	0.6352 (11)	0.109 (4)	0.322 (16)
O4	0.3901 (18)	0.161 (3)	0.7605 (10)	0.163 (9)	0.322 (16)
O1'	0.2309 (8)	0.0465 (8)	0.7085 (4)	0.100 (2)	0.678 (16)
O2'	0.2776 (6)	0.2018 (6)	0.6061 (3)	0.0944 (18)	0.678 (16)
O3'	0.4372 (9)	0.0699 (10)	0.6589 (6)	0.133 (3)	0.678 (16)
O4'	0.3540 (11)	0.2203 (8)	0.7502 (5)	0.125 (3)	0.678 (16)
O1WA	0.559 (2)	0.3844 (18)	0.6839 (13)	0.097 (5)	0.322 (16)
H1WA	0.543 (12)	0.457 (6)	0.702 (10)	0.146*	0.322 (16)
H2WA	0.493 (7)	0.342 (10)	0.676 (11)	0.146*	0.322 (16)
O1WB	0.6081 (9)	0.3409 (9)	0.6947 (5)	0.089 (2)	0.678 (16)
H1WB	0.620 (6)	0.387 (5)	0.739 (3)	0.134*	0.678 (16)
H2WB	0.549 (5)	0.292 (5)	0.703 (4)	0.134*	0.678 (16)
O6	0.85711 (15)	0.49047 (14)	0.30449 (8)	0.0523 (4)	
N4	0.84030 (15)	0.52739 (15)	0.45051 (9)	0.0389 (4)	
N5	0.68242 (16)	0.41812 (16)	0.52703 (11)	0.0481 (4)	
H5	0.6483	0.4026	0.5768	0.058*	
N6	0.82368 (19)	0.55766 (18)	0.59950 (11)	0.0582 (5)	
H6A	0.8880	0.6097	0.5994	0.070*	
H6B	0.7866	0.5420	0.6482	0.070*	
C6	0.78157 (18)	0.50162 (17)	0.52456 (12)	0.0410 (4)	
C7	0.79858 (19)	0.46791 (18)	0.37411 (12)	0.0417 (4)	
C8	0.6914 (2)	0.3822 (2)	0.37529 (14)	0.0538 (5)	
H8	0.6605	0.3434	0.3230	0.065*	
C9	0.6351 (2)	0.3576 (2)	0.45156 (14)	0.0508 (5)	
C10	0.5254 (3)	0.2665 (3)	0.46219 (18)	0.0743 (7)	
H10A	0.4962	0.2344	0.4050	0.111*	
H10B	0.4558	0.3103	0.4890	0.111*	
H10C	0.5538	0.1963	0.4994	0.111*	

### Atomic displacement parameters (Å^2^)

**Table:**

	*U*^11^	*U*^22^	*U*^33^	*U*^12^	*U*^13^	*U*^23^
O5	0.0660 (9)	0.0544 (8)	0.0244 (6)	−0.0120 (7)	0.0079 (6)	−0.0008 (5)
N1	0.0466 (9)	0.0390 (8)	0.0257 (7)	−0.0069 (6)	0.0037 (6)	−0.0001 (6)
N2	0.0459 (9)	0.0422 (8)	0.0281 (7)	−0.0032 (6)	0.0066 (6)	0.0057 (6)
N3	0.0691 (11)	0.0525 (9)	0.0256 (7)	−0.0143 (8)	0.0069 (7)	0.0002 (7)
C1	0.0412 (10)	0.0392 (9)	0.0269 (8)	0.0025 (7)	0.0041 (7)	0.0035 (7)
C2	0.0454 (10)	0.0436 (9)	0.0264 (8)	−0.0001 (8)	0.0039 (7)	−0.0011 (7)
C3	0.0527 (12)	0.0493 (10)	0.0335 (9)	−0.0091 (9)	0.0005 (8)	−0.0039 (8)
C4	0.0415 (10)	0.0420 (9)	0.0388 (9)	−0.0020 (8)	0.0030 (8)	0.0032 (8)
C5	0.0631 (14)	0.0588 (13)	0.0523 (12)	−0.0214 (11)	0.0065 (10)	0.0013 (10)
Cl1	0.0656 (4)	0.0782 (4)	0.0458 (3)	−0.0041 (3)	0.0098 (3)	−0.0022 (3)
O1	0.053 (5)	0.129 (10)	0.116 (11)	−0.008 (5)	0.037 (5)	−0.013 (7)
O2	0.25 (2)	0.100 (10)	0.28 (2)	0.081 (11)	0.129 (16)	0.109 (12)
O3	0.064 (6)	0.143 (8)	0.120 (8)	0.019 (5)	0.000 (5)	−0.057 (7)
O4	0.125 (11)	0.28 (3)	0.081 (7)	−0.063 (14)	−0.040 (7)	−0.040 (12)
O1'	0.147 (6)	0.103 (4)	0.050 (2)	−0.066 (4)	0.023 (3)	−0.003 (2)
O2'	0.111 (3)	0.114 (4)	0.058 (2)	0.019 (3)	0.005 (2)	0.025 (2)
O3'	0.090 (5)	0.173 (7)	0.137 (6)	0.060 (5)	0.026 (4)	0.024 (4)
O4'	0.145 (6)	0.139 (5)	0.091 (4)	−0.052 (4)	0.010 (3)	−0.053 (3)
O1WA	0.117 (12)	0.095 (10)	0.083 (9)	−0.046 (8)	0.044 (9)	−0.025 (7)
O1WB	0.108 (5)	0.096 (5)	0.063 (2)	−0.035 (3)	0.008 (3)	0.013 (3)
O6	0.0736 (10)	0.0537 (8)	0.0303 (7)	−0.0154 (7)	0.0105 (7)	−0.0060 (6)
N4	0.0467 (9)	0.0413 (8)	0.0292 (7)	−0.0049 (7)	0.0057 (6)	−0.0030 (6)
N5	0.0461 (9)	0.0579 (10)	0.0412 (8)	−0.0084 (8)	0.0100 (7)	0.0035 (7)
N6	0.0717 (12)	0.0730 (12)	0.0312 (8)	−0.0255 (10)	0.0160 (8)	−0.0090 (8)
C6	0.0465 (10)	0.0425 (9)	0.0346 (9)	−0.0010 (8)	0.0076 (8)	−0.0003 (7)
C7	0.0500 (11)	0.0432 (10)	0.0321 (8)	−0.0030 (8)	0.0029 (8)	−0.0029 (7)
C8	0.0576 (13)	0.0617 (12)	0.0415 (10)	−0.0159 (10)	−0.0029 (9)	−0.0029 (9)
C9	0.0449 (11)	0.0559 (11)	0.0513 (11)	−0.0096 (9)	−0.0010 (9)	0.0020 (10)
C10	0.0633 (16)	0.0858 (18)	0.0738 (16)	−0.0298 (13)	0.0041 (13)	0.0066 (14)

### Geometric parameters (Å, °)

**Table:**

O5—C2	1.233 (2)	Cl1—O1	1.408 (10)
N1—C1	1.347 (2)	Cl1—O1'	1.419 (5)
N1—C2	1.393 (2)	O1WA—H1WA	0.82 (2)
N1—H1	0.8600	O1WA—H2WA	0.82 (2)
N2—C1	1.335 (2)	O1WB—H1WB	0.831 (19)
N2—C4	1.373 (2)	O1WB—H2WB	0.82 (2)
N2—H2	0.8600	O6—C7	1.260 (2)
N3—C1	1.311 (2)	N4—C6	1.325 (2)
N3—H3A	0.8600	N4—C7	1.361 (2)
N3—H3B	0.8600	N5—C6	1.349 (2)
C2—C3	1.421 (3)	N5—C9	1.371 (3)
C3—C4	1.353 (2)	N5—H5	0.8600
C3—H3	0.9300	N6—C6	1.327 (2)
C4—C5	1.496 (3)	N6—H6A	0.8600
C5—H5A	0.9600	N6—H6B	0.8600
C5—H5B	0.9600	C7—C8	1.427 (3)
C5—H5C	0.9600	C8—C9	1.339 (3)
Cl1—O2	1.369 (10)	C8—H8	0.9300
Cl1—O4	1.373 (10)	C9—C10	1.497 (3)
Cl1—O4'	1.395 (5)	C10—H10A	0.9600
Cl1—O3	1.396 (10)	C10—H10B	0.9600
Cl1—O3'	1.397 (6)	C10—H10C	0.9600
Cl1—O2'	1.398 (4)		
			
C1—N1—C2	123.33 (15)	O2—Cl1—O1	110.4 (9)
C1—N1—H1	118.3	O4—Cl1—O1	109.0 (9)
C2—N1—H1	118.3	O3—Cl1—O1	106.8 (8)
C1—N2—C4	122.40 (14)	O4'—Cl1—O1'	108.8 (4)
C1—N2—H2	118.8	O3'—Cl1—O1'	110.3 (4)
C4—N2—H2	118.8	O2'—Cl1—O1'	109.8 (4)
C1—N3—H3A	120.0	H1WA—O1WA—H2WA	111 (4)
C1—N3—H3B	120.0	H1WB—O1WB—H2WB	109 (3)
H3A—N3—H3B	120.0	C6—N4—C7	118.73 (15)
N3—C1—N2	121.65 (15)	C6—N5—C9	121.13 (16)
N3—C1—N1	119.85 (16)	C6—N5—H5	119.4
N2—C1—N1	118.50 (15)	C9—N5—H5	119.4
O5—C2—N1	118.64 (16)	C6—N6—H6A	120.0
O5—C2—C3	125.68 (16)	C6—N6—H6B	120.0
N1—C2—C3	115.68 (15)	H6A—N6—H6B	120.0
C4—C3—C2	120.48 (17)	N4—C6—N6	118.84 (17)
C4—C3—H3	119.8	N4—C6—N5	122.41 (16)
C2—C3—H3	119.8	N6—C6—N5	118.74 (16)
C3—C4—N2	119.50 (16)	O6—C7—N4	118.29 (16)
C3—C4—C5	124.79 (18)	O6—C7—C8	122.28 (17)
N2—C4—C5	115.70 (16)	N4—C7—C8	119.43 (16)
C4—C5—H5A	109.5	C9—C8—C7	120.16 (19)
C4—C5—H5B	109.5	C9—C8—H8	119.9
H5A—C5—H5B	109.5	C7—C8—H8	119.9
C4—C5—H5C	109.5	C8—C9—N5	118.10 (18)
H5A—C5—H5C	109.5	C8—C9—C10	125.3 (2)
H5B—C5—H5C	109.5	N5—C9—C10	116.56 (19)
O2—Cl1—O4	111.9 (9)	C9—C10—H10A	109.5
O2—Cl1—O3	109.9 (9)	C9—C10—H10B	109.5
O4—Cl1—O3	108.7 (8)	H10A—C10—H10B	109.5
O4'—Cl1—O3'	109.8 (5)	C9—C10—H10C	109.5
O4'—Cl1—O2'	109.8 (4)	H10A—C10—H10C	109.5
O3'—Cl1—O2'	108.4 (4)	H10B—C10—H10C	109.5
			
C4—N2—C1—N3	177.34 (17)	C7—N4—C6—N6	178.73 (18)
C4—N2—C1—N1	−1.9 (3)	C7—N4—C6—N5	0.4 (3)
C2—N1—C1—N3	179.95 (17)	C9—N5—C6—N4	−1.6 (3)
C2—N1—C1—N2	−0.8 (3)	C9—N5—C6—N6	179.98 (19)
C1—N1—C2—O5	−177.23 (18)	C6—N4—C7—O6	−178.20 (18)
C1—N1—C2—C3	3.4 (3)	C6—N4—C7—C8	1.5 (3)
O5—C2—C3—C4	177.18 (19)	O6—C7—C8—C9	177.6 (2)
N1—C2—C3—C4	−3.4 (3)	N4—C7—C8—C9	−2.1 (3)
C2—C3—C4—N2	1.1 (3)	C7—C8—C9—N5	0.9 (3)
C2—C3—C4—C5	−179.47 (19)	C7—C8—C9—C10	−177.8 (2)
C1—N2—C4—C3	1.8 (3)	C6—N5—C9—C8	1.0 (3)
C1—N2—C4—C5	−177.75 (17)	C6—N5—C9—C10	179.7 (2)

### Hydrogen-bond geometry (Å, °)

**Table:**

*D*—H···*A*	*D*—H	H···*A*	*D*···*A*	*D*—H···*A*
O1WA—H2WA···O2	0.82 (2)	2.26 (6)	3.07 (4)	169 (16)
C5—H5B···O3'	0.96	2.55	3.509 (6)	173.
N5—H5···O1WB	0.86	1.95	2.797 (8)	166.
O1WB—H2WB···O4'	0.82 (2)	2.30 (4)	3.071 (14)	159 (6)
N1—H1···N4^i^	0.86	1.98	2.839 (2)	174.
N3—H3B···O6^i^	0.86	1.93	2.787 (2)	178.
N6—H6A···O5^i^	0.86	2.05	2.895 (2)	168.
N2—H2···O6^ii^	0.86	1.84	2.6560 (18)	158.
N3—H3A···O5^ii^	0.86	2.24	3.0363 (18)	154.
N6—H6B···O1^iii^	0.86	2.35	3.095 (17)	145.
C3—H3···O1^iv^	0.93	2.56	3.466 (17)	166.

Symmetry codes: (i) −*x*+2, −*y*+1, −*z*+1; (ii) *x*, −*y*+1/2, *z*+1/2; (iii) −*x*+1, *y*+1/2, −*z*+3/2; (iv) −*x*+1, −*y*, −*z*+1.
